# Halogen Bonding in (*Z*)-2-Iodocinnamaldehyde

**DOI:** 10.3390/molecules18088712

**Published:** 2013-07-24

**Authors:** Pakorn Bovonsombat, Francesco Caruso, Andrew Jdaydani, Miriam Rossi

**Affiliations:** 1 Mahidol University International College, Mahidol University, Salaya Campus, Nakorn Pathom 73170, Thailand; 2 Istituto Chimica Biomolecolare CNR, P.le Aldo Moro 5, Rome 00185, Italy; 3 Department of Chemistry, Vassar College, Poughkeepsie, NY 12604-0484, USA

**Keywords:** halogen bond, α-iodoenals, crystal structure, DFT

## Abstract

Based on the bulkiness of the iodine atom, a non-planar conformation was expected for the title compound. Instead, its molecular structure is planar, as experimentally determined using single crystal X-ray diffraction, and confirmed theoretically by DFT calculations on the single molecule and the halogen pair paired molecules, therefore ruling out crystal packing forces as a principal factor leading to planarity. Indeed, planarity is ascribed to the carbonyl double bond, as when this bond is saturated on forming the related alcohol derivative, the molecule loses planarity. The X-ray molecular structure shows an intermolecular separation between the iodine and the oxygen of the carbonyl shorter than the corresponding van der Waals distance suggesting a weak halogen bond interaction. DFT minimization of this 2-molecule arrangement shows the iodine--oxygen distance much shorter than that observed in the crystal interaction and confirming its stronger halogen bond nature. A trend between increasing I•••O(carbonyl) separation and decreasing C-I•••O(carbonyl) angle is demonstrated, further confirming the existence of a halogen bond.

## 1. Introduction

The iodo moiety in α-iodo-enals and -enones is the preferred halogen in the asymmetric synthesis of several important biologically-active compounds such as (−)-brunsvigine [1], (−)-manthine [1], (−)-strychnine [2], shikimic acid analogues [3], fluoroneplanocin A [4] and (−)-tetrodotoxin [5]. Due to the ease, compared to other halogens, with which it undergoes carbon-carbon coupling, the iodine of α-iodo-enals and -enones is also the preferred halogen substrate of choice in the Stille, Heck and Suzuki Pd-catalysed coupling reactions [6,7,8,9,10]. During the characterization of one of these aldehydes synthesized by our group, which involved a determination of the X-ray crystal structure of the title compound [(*Z*)-2-iodocinnamaldehyde], an intermolecular halogen interaction C−I•••O=C was seen and is reported herein.

The definition of halogen bonds, shares many features with that of hydrogen bonds: they are attractive noncovalent interactions occurring when a covalently bound electron-deficient halogen atom pairs with nearby Lewis bases, in our case the carbonyl oxygen atom. Computational studies have shown that halogen bound organic donors produce weaker halogen bonds that are more electrostatic in nature [11]. Evidence for a weak electrostatic interaction between the positively polarized iodine atom and the nucleophilic carbonyl O atom is seen, experimentally, from the single crystal X-ray structure, and in calculations using DFT methods. The I•••O distance is less than the sum of the van der Waals radii for iodine and oxygen and the C−I•••O angle [149.33(5)°] suggest a weak halogen bond in our molecule.

## 2. Results and Discussion

### 2.1. Chemistry

The direct synthesis of α-iodoenones or α-iodo-α,β-unsaturated ketones from enones is well established [12,13,14,15,16,17,18,19,20,21,22]. However, examples of direct synthesis of linear α-iodo-α,β-unsaturated aldehydes (α-iodoenals) from enals are scarce and only two, those of (*Z*)-2-iodobutenal [23] and (*Z*)-2-iodocinnamaldehyde [24], are known thus far. 

**Scheme 1:**
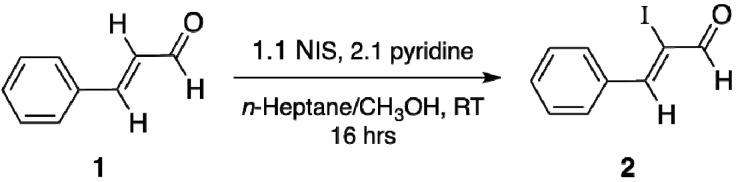
Synthesis of **2** via direct α-iodination of *trans*-cinnamaldehyde.

**Figure 1:**
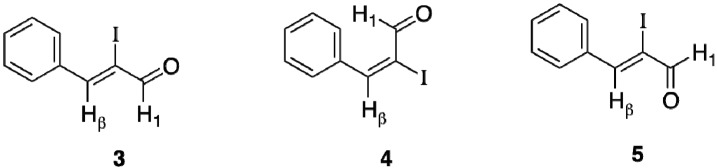
Isomers and conformations of **2**.

For the synthesis of (*Z*)-2-iodocinnamaldehyde (**2**), a published procedure for the synthesis of (*Z*)-2-bromocinnamaldehyde was adopted [24] (Scheme 1). *N*-iodosuccinimide (NIS) was employed as the source of iodine in the reaction, analogous to *N*-bromosuccinimide acting as the bromine donor in the synthesis of (*Z*)-2-bromocinnamaldehyde. In the iodination of *trans*-cinnamaldehyde (**1**), pyridine was more effective than pyridine-*N*-oxide and was utilised in the *n*-heptane-methanol solvent system, which consistently gave high yields of **2** (52% isolated yield) compared to those obtained from a single organic solvent system. The specificity for the *Z*-isomer is extremely high, with a *Z*/*E* isomer ratio, as determined by GC-MS, of 405:1. Evidence for iodination at the α-position of the enal comes from the absence of α-hydrogen ^1^H-NMR absorptions in compound **2**. Doublets, observed for the aldehyde hydrogens in **1** (caused by coupling of the α-geminal vinyl hydrogen), were also absent in **2**. These were instead replaced by singlets, which would be consistent with substitution of the α-hydrogen by an iodine atom. The splitting pattern of the β-hydrogens of **2** was less complicated, becoming a triplet (due to the *trans* α-hydrogen and the allyl hydrogen couplings). Nevertheless, iodo substitution at the α-position can still furnish two geometric isomers, the *Z*-isomer **3**, shown in an *s*-*trans* conformation, and the *E*-isomer **4**. Furthermore, **3** can also exist in another conformation, the *s*-*cis* (**5**) (Figure 1). 

NOE experiments were performed to establish both the geometries and the conformations of **2**. Irradiation of the β-hydrogen (H_β_) of **2** gave a 6.9% intensity enhancement of the aldehyde hydrogen (H_1_), and an 8.0% intensity enhancement for H_β_ was observed, when H_1_ was irradiated. The intensity enhancements of H_1_ and H_β_, when either was irradiated, are consistent with structure **3** thereby confirming the geometries of **2** as *Z*. In contrast, the geometric isomer **4** is not expected to show any intensity enhancements when either H_1_ or H_β_ is irradiated. Furthermore, the observed NOE effects firmly established that the conformation of **2** is *s*-*trans*, which would be consistent with **3** and not **5**.

### 2.2. Single Crystal X-ray Diffraction Study

**Figure 2:**
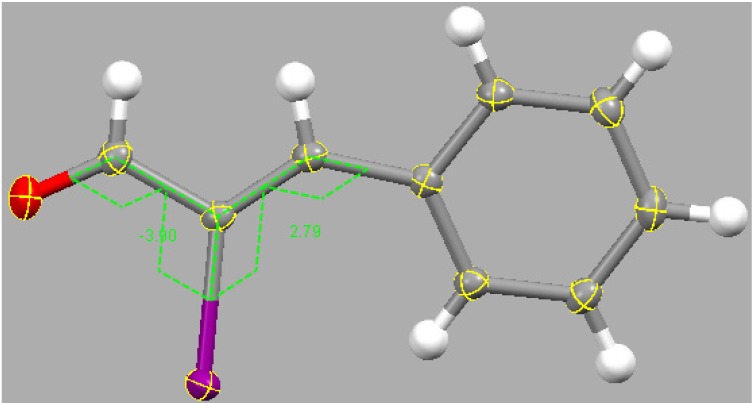
X-ray molecular structure of the title compound including the two torsion angles, −3.9° and 2.8° surrounding the iodine. These values, although small, are greater than the unsubstituted cinnamaldehyde [26] (1.06° and 0.85°, respectively) due to the bulky iodine atom.

The X-ray structure of (*Z*)-2-Iodocinnamaldehyde shows the *s*-trans conformation of the enal structure, confirming the aforementioned COSY study and a wealth of information available on the conformation of enals (and enones) [25]. The almost planar molecular structure is depicted in Figure 2; the torsion angles O-C-C-I [−3.9(2)°] and C-C-C-I [2.8(3)°] are close to 0°, whereas the iodo-propenal fragment is out of the plane of the benzene ring, C-C-C-H torsion angle 10.35(3)° and similar to the unhalogenated cinnamaldehyde [26] C-C-C-H torsion angle 9.36(2)°. Also, we see a long C(aryl)–C(sp^2^) 1.464(2) Å similar to that seen in [26] (1.466(2) Å) and indicating in both cases, poor resonance between the propenal fragment and the aromatic ring. The C-I bond length 2.085(2) Å is expected for a C(sp^2^)-I, according to values seen in CSD. In [26] the carbonyl O atom has weak, 3.304(2) Å, hydrogen bond with the *para*-H—C(aryl).

**Figure 3:**
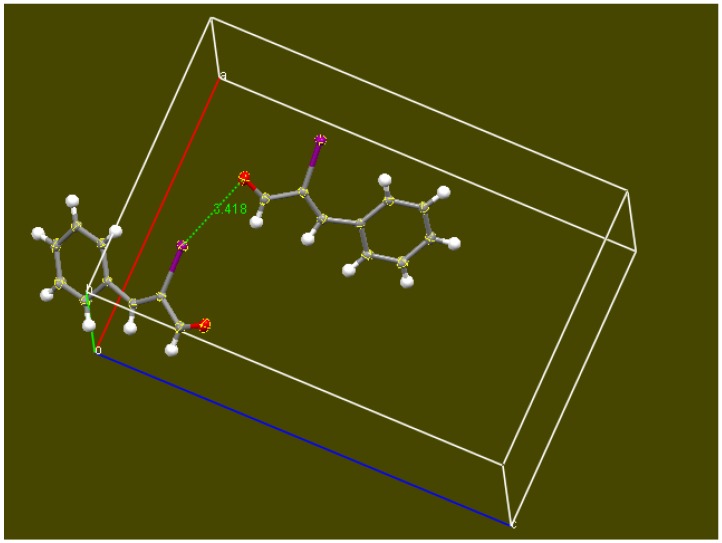
Intermolecular I--O separation (3.418 Å) in the crystal, non-hydrogen atoms shown with their ellipsoids; C-I•••O(carbonyl) = 149.33(5)°.

Figure 3 shows two out of eight molecules in the unit cell and an interaction between the iodine and the O(carbonyl) is seen. The I•••O separation of 3.418(2) Å is shorter than the sum of the O and I van der Waals radii of 3.50 Å, indicating a potential halogen bond. To exclude the possibility of this feature resulting from packing forces which could constrain the molecules to be closer than expected, a theoretical study was carried out. 

### 2.3. Theoretical Study

It is known that in a C-X bond, the halogen atom often acts as a Lewis acid since positive electrostatic potential is observed on it and directly opposite to the C-X bond (the σ-hole). An example has been described for CF_3_Br [27]. 

**Figure 4:**
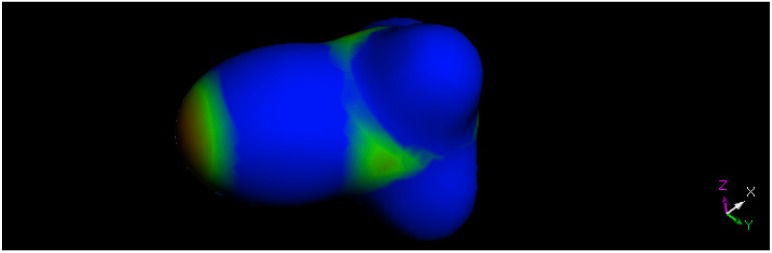
CF_3_Br electrostatic potential, obtained after its geometry optimization, and using the same conditions for the title compound; the Br atom is located on the left, only two F atoms are visible on the right, and the C atom is seen as green shaded in the center. Color range blue-green-red (more negative (blue) …green…less negative (red)).

To verify the ability of Dmol^3^ to describe halogen bonds, we performed an *ab-initio* geometry optimization on a CF_3_Br molecule and obtained its electrostatic potential, as depicted in Figure 4. The area on the bromine atom but opposite to the C-Br bond (left part) appears positively charged (red color), consistent with published results. 

**Figure 5:**
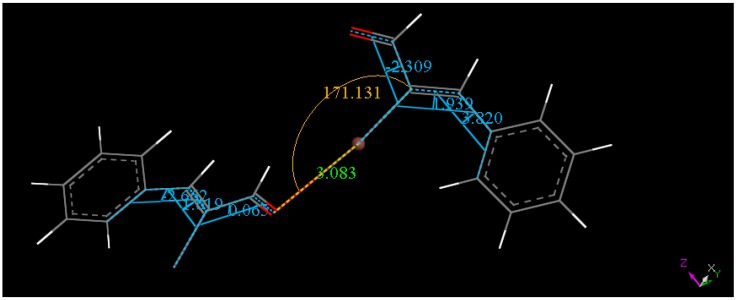
After geometry optimization of the two-molecule arrangement in the crystal, the resulting DFT minimum arrangement is shown; green distance, yellow angle and light blue torsion angles.

In our case, two molecules, as shown in Figure 3, were input in Dmol^3^ program and geometrically optimized. The converged system depicted in Figure 3 is at a minimum of energy. It is clear that an attractive interaction is present between both molecules; otherwise the geometry optimization would separate the molecules. In addition, this 2-molecule calculated arrangement displays a I•••O separation much shorter than in the crystal [3.083 Å *vs.* 3.418(2) Å, respectively]. This suggests the packing in the crystal is not responsible for the short I•••O intermolecular distance. Furthermore, halogen bonds display strong directionality according to Resnati and Metrangolo [28], and so we expect the O•••I-C angle to be close to 180°, as confirmed in Figure 5. 

Comparing energies of two single molecules and the two-molecule arrangement of Figure 3, the latter is favored by 4.6 kcal/mol. An additional calculation included a different two-molecule arrangement found in the crystal where I and O(carbonyl) were not related by intermolecular interactions; this arrangement was also more stable than two times the single molecule (3.4 kcal/mol) but not as much as the previous one.

**Figure 6:**
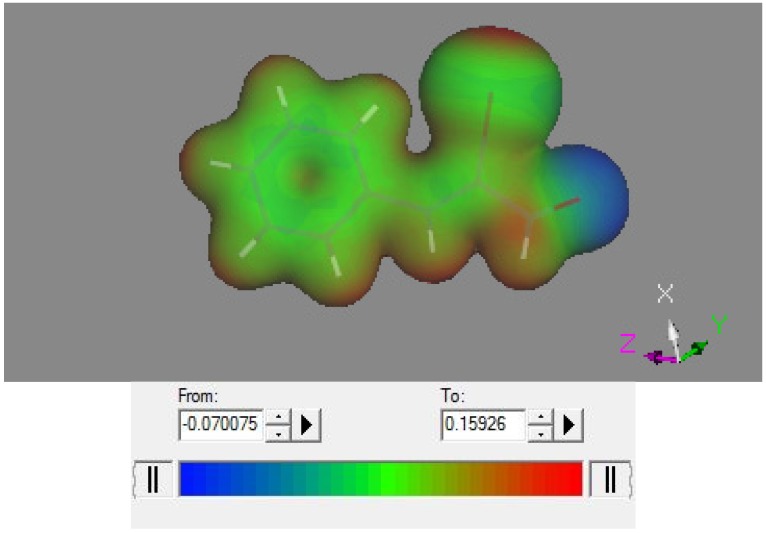
Electrostatic potential for the title compound in the crystal, the iodine atom is located in the upper right area and clearly shows and positive (red) σ-hole.

**Figure 7:**
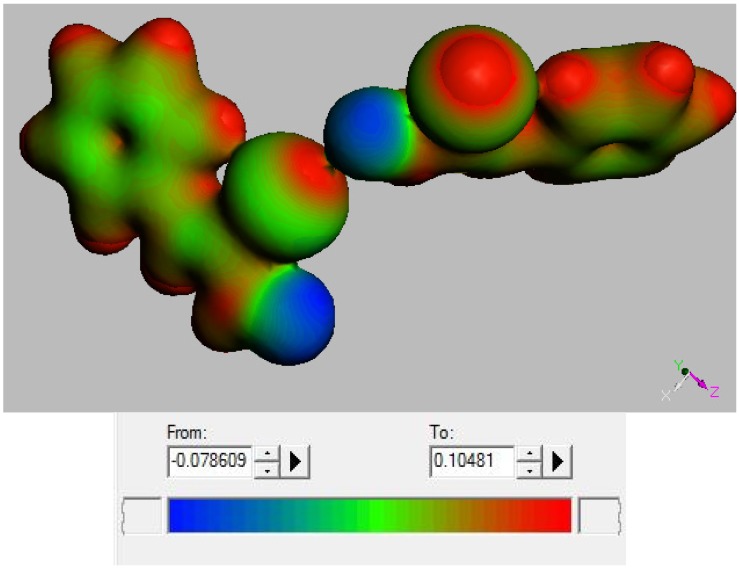
Electrostatic potential for the two-molecule arrangement in the crystal (also shown in Figure 3).

The single molecule, and the two-molecule arrangement (both as found in the crystal), were input in Dmol^3^ and the electrostatic potential calculated. Results are depicted in Figure 6 and Figure 7 and appear consistent with earlier studies on halogen bonded compounds.

To further detect a halogen bond in the title compound we performed an additional calculation on the two-molecule arrangement. We selected and fixed several C-I•••O(carbonyl) angles and performed geometry optimization calculations. For a C-I•••O(carbonyl) angle of about 140º the I•••O(carbonyl) separation becomes 3.323 Å, which is longer than that of the real minimum (171.1°, 3.083 Å). For a fixed C-I•••O(carbonyl) angle of about 120°, the I•••O(carbonyl) separation (3.595 Å) increases and is slightly longer than the van der Waals separation (3.5 Å), whereas a fixed C-I•••O(carbonyl) angle of about 90° results in even longer (3.862 Å) I•••O(carbonyl) separation, suggesting both molecules drifting apart. Since halogen bonds, like hydrogen bonds, are more effective with angles about 180° and absent for small angles, these results are consistent with halogen bond existence in the title compound.

In addition, in this series of calculation the smaller the angle the higher the energy, which indicates more stability for the non-fixed angle minimization (C-I•••O(carbonyl) angle = 171.1°, Figure 5). Additional calculations were performed on the crystal, the cell was minimized using the same bases and functional. Variation in cell dimensions are found, a = 11.262 Ǻ (11.473 Ǻ, Xray); b = 8.849 Ǻ (8.674 Ǻ Xray) c = 16.316Ǻ (16.827 Å, Xray). The calculated shrunken cell makes the I•••O(carbonyl) separation shorter (3.347 Å) than in the crystal, even shorter than van der Waals separation. We also calculated the lattice energy whose value is −27.7 kcal/mol.

**Figure 8:**
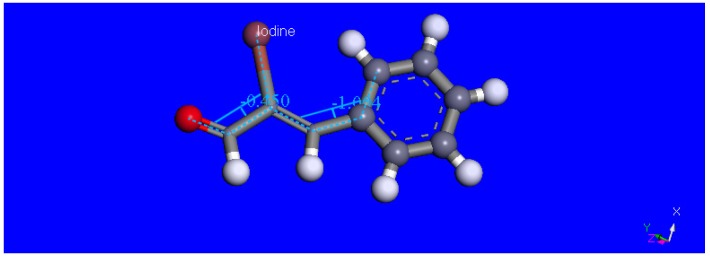
The geometrically optimized title compound.

**Figure 9:**
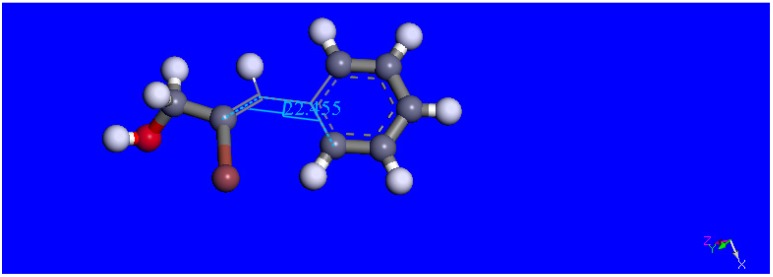
The geometrically optimized alcohol derivative, showing lack of co-planarity between the Ph ring and C=C double bond (torsion angle = 22.45°).

As described above, the crystal structure of the title compound shows marked co-planarity in the molecule, in spite of the bulky iodine atom that might induce steric hindrance with the *ortho*-hydrogens of the phenyl ring. The question arises whether this co-planarity of the phenyl ring (with respect to the enal moiety) is due to both the carbonyl and the alkene, or is one of them sufficient to overcome the hindrance posed by the iodine? It seems obvious that saturating the alkene moiety should remove the co-planarity, but a carbonyl variation might disrupt the conjugation of the phenyl ring to the enal. In order to answer this question, a theoretical study on a variation of the carbonyl, the alcohol derivative, was undertaken. Figure 8 shows the co-planar DFT geometry optimized minimum carbonyl species, whereas the alcohol derivative optimized structure, Figure 9, loses co-planarity (torsion angle = 22.4°). Therefore, the effect of co-planarity is driven by the carbonyl moiety, which is an interesting example of long-range structural influence. The co-planarity finding is consistent with that described by Liljefors and Allinger [29], which showed enals possessing co-planarity conformation, even for those enals with methyl groups located geminal to the aldehyde or *cis* to the aldehyde. The calculations done by Liljefors and Allinger using molecular method (VESCF) predicted these enals to have an aldehyde-alkene dihedral angle of 180°. Furthermore the co-planar finding from the crystal structure of **2** is also consistent with the dipole study [30] and the findings from spectroscopic studies [31,32,33,34].

Our DFT optimized I--O interaction in the 2-molecule arrangement can be compared with that observed in the crystal structure [35] of methyl-2-chloro-5-iodo-4-(2-(1-(ethoxycarbonyl)ethylidene)-hydrazino)benzoate [d(C=O--I) = 3.073(8) Å, O--I-C = 179.9(8)°]. 

## 3. Experimental

### 3.1. Chemistry

*trans*-Cinnamaldehyde was obtained from Aldrich (St. Louis, MO, USA) and pyridine was from Fluka (Buchs, Switzerland). Acros Organics (Geel, Belgium) was the supplier of *N*-iodosuccinimide (NIS). The reagents were used without further purification. Methanol and n-heptane used in the reactions are of AR grade and were from RCI LabScan Co. Ltd. (Bangkok, Thailand). ^1^H- and ^13^C-NMR spectra were recorded on a Bruker Avance 300 MHz spectrometer in CDCl_3_ using TMS as an internal standard. The products composition and relative yields were carried out on a gas chromatograph-mass spectrometer (Agilent 6890 GC system and Agilent 5973 Mass Selective Detector) using HP-1 capillary column (0.32 mm × 24.9 m × 017 μm). IR spectra were recorded on a Perkin–Elmer Spectrum 100 FT-IR Spectrometer. Separations of products were carried out on a centrifugal thin-layer chromatography (Harrison Research, Palo Alto, CA, USA) using a plate coated with 2 mm of silica gel 60GF_254_. 

### Synthesis Procedure of (Z)-2-iodo-3-phenylpropenal (**2**).

*trans*-Cinnamaldehyde (63 μL, 0.5 mmol) was dissolved in a methanol (0.5 mL)-*n*-heptane (9.5 mL) solvent system, containing pyridine (85 μL, 1.04 mmol, 2.1 equivs.). NIS (0.1284 g, 0.56 mmol) was then added in one portion to the solution and stirred at room temperature for 16 hours. The mixture was then diluted with Et_2_O (60 mL) and washed with 10% sodium thiosulfate (3 × 20 mL), H_2_O (2 × 20 mL) and brine (2 × 10 mL). The combined aqueous solution was re-extracted with Et_2_O (10 mL). The combined Et_2_O solution was dried over anhydrous Na_2_SO_4_. Purification of the iodo product was conducted with silica gel chromatography using 2% CH_2_Cl_2_/hexanes as the eluent. 

*Compound* **2**: light yellow solid (0.067 g, 52% purified yield); mp 88–89 °C (lit. [24] mp 89–90 °C); ^1^H NMR (300 MHz, CDCl_3_): δ 7.44–7.57 (m, 3H, aromatic C-3, C-4 and C-5 *H*s), 7.95–8.05 (m, 2H, aromatic C-2 and C-6 *H*s), 8.10 (s, 1H, C*H*=CI, NOE, enhancement from CHO, 7.9%), 8.79 (s, 1H, C*H*O, NOE, enhancement from β-H, 6.9%); ^13^C-NMR (75 MHz, CDCl_3_): δ 105.9, 128.6, 130.4, 131.6, 134.0, 155.8, 189.0; GC-MS (EI), *m/z* (rel int.): 259 (10, (M+1)^+^), 258 (100, M^+^), 257 (43, (M-1)^+^), 131 (25, (M-I)^+^), 130 (35, (M-HI)^+^), 127 (5, I), 103 (76, ((M+1)-CHO)^+^), 102 (54, (M-CHO)^+^), 77 (60, C_6_H_5_), 51 (22); IR (ATR): 1669, 1612 cm^−1^.

### 3.2. X-ray Diffraction Study

Suitable crystals for X-ray diffraction of the title compound were obtained from an ether/hexane (3:7) solution at room temperature after about a week. Diffraction data were collected at 125K using a Bruker SMART APEX II CCD X-ray diffractometer. Structure resolution and refinement were performed using SHELXTL [36]; details are included in Table 1. H atoms not found in Fourier maps were included from models and constrained as riding on their bound atoms. CCDC 940095 contains the supplementary crystallographic data for this paper. These data can be obtained free of charge via www.ccdc.cam.ac.uk/conts/retrieving.html (or from the CCDC, 12 Union Road, Cambridge CB2 1EZ, UK; fax: +44 1223 336033; e-mail: deposit@ccdc.cam.ac.uk).

**Table 1:** Crystal data and refinement details of (*Z*)-2-iodocinnamaldehyde.

Empirical formula	C_9_H_7_INO
Crystal color	colorless
Formula weight	258.05
Crystal System	Orthorhombic
Space group	P bca
Temperature K	125(2)
Wavelength (Å)	0.71073
*a* (Å)	11.4372(6)
*b* (Å)	8.6736(5)
*c* (Å)	16.8274(9)
Volume (Å^3^)	1669.31(16)
Z, density (mg/mm^3^)	8, 2.053
Absorption coefficient	3.772
Crystal size (mm)	0.25 × 0.17 × 0.10
θ range data collection	2.42, 30.03
Limiting índices	−16,16/−12.12/−23,23
Data collected /unique	24123, 2436
Max, min. Transmission	0.45/0.70
Refinement method	F^2^
Refined data / parameters	2057/128
Goodness-of-fit on F^2^	1.026
Final R, Rw [I > 2sigma(I)]	0.0181/0.0408

### 3.3. DFT Study

Calculations were done using commercial software programs from Accelrys [37]. Density functional theory (DFT) code DMol3 was applied to calculate geometries, energies, and frequencies, implemented in Materials Studio 6.1, using a PC platform [38]. We employed Double Numerical Polarized (DNP) basis set that includes all the occupied atomic orbitals plus a second set of valence atomic orbitals plus polarized d-valence orbitals [39], and correlation generalized gradient approximation (GGA) was applied in the manner (PBE) suggested by Perdew-Burke-Ernzerhof [40]; the recent inclusion of a dispersion term that deals with O•••H interactions was also applied (GRIMME) [41,42].

Spin unrestricted approach was exploited with all electrons being considered explicitly. The real space cutoff of 6 Å was imposed for numerical integration of the Hamiltonian matrix elements. The self-consistent-field convergence criterion was set to the root-mean square change in the electronic density to be less than 10^−6^ electron/Å^3^. The convergence criteria applied during geometry optimization were 2.72 10^−4^ eV for energy and 0.054 eV/Å for force. 

## 4. Conclusions

Based on the bulkiness of the iodine atom and the potential steric hindrance posed on the *ortho*-hydrogens of the phenyl ring, the molecular structure of the title compound was expected to be non-planar. X-ray diffraction results demonstrated instead that the related torsion angles were close to 0°. Packing forces were not responsible for co-planarity as confirmed by DFT calculations, which also show molecular co-planarity. The driving force explaining co-planarity is assigned to the carbonyl double bond, as when it is saturated, forming the related alcohol derivative (replacing HC=O by H_2_COH), the molecule loses co-planarity. The X-ray molecular structure shows an intermolecular separation between the iodine and the oxygen of the carbonyl shorter than the corresponding van der Waals distance. DFT minimization of the molecular arrangement of the title compound makes the I•••O interaction stronger as its distance is shorter than in the crystal, confirming its halogen bond nature.

## Supplementary Materials

Supplementary materials can be accessed at: http://www.mdpi.com/1420-3049/18/8/8712/s1.
